# Fatal transfusion related acute lung injury following coronary artery by-pass surgery: a case report

**DOI:** 10.1186/1757-1626-1-372

**Published:** 2008-12-03

**Authors:** Fauzia Ahmad Bawany, Hasanat Sharif

**Affiliations:** 1 Section of Cardiothoracic Surgery, Department of Surgery, Aga Khan University, Stadium Road, Karachi 74800, Pakistan

## Abstract

**Background:**

Transfusion related acute lung injury (TRALI) is a potentially fatal Acute Lung Injury following transfusion of blood components. Hypotheses implicate donor-derived anti-human leukocyte antigen or granulocyte antibodies reacting with recipients' leukocytes, releasing inflammatory mediators. Lack of agreement on underlying cellular and molecular mechanisms renders improving transfusion safety difficult and expensive.

**Case Presentation:**

Literature search has not revealed any case of TRALI from Pakistan. We report the case of fatal TRALI in a 68 year old male who received blood products after coronary artery by-pass surgery.

**Conclusion:**

This article aims to create awareness about this complication and suggests that post transfusion cardiopulmonary instability should alert to the possibility of TRALI.

## Background

TRALI is a potentially fatal complication of transfusion occurring in 1 per 5000[1]. However, being an under recognized and under-reported condition, information about TRALI is mostly patchy and the true global incidence is unknown. To the best of our knowledge, this is the first ever documented case of TRALI from Pakistan.

## Case presentation

A 68-year previously healthy, retired male presented with inferior wall myocardial infarction. Cardiac catheterization revealed critical three-vessel Coronary Artery Disease (CAD) for which he received streptokinase and was admitted for Coronary Artery By-pass Graft (CABG) surgery.

His weight was 69 kg, height 162 cm, and he was of Asian origin. He was a non-smoker with no associated co-morbid conditions. He had a family history of diabetes mellitus (father). Pre-operative evaluation included a normal chest radiograph and unremarkable routine blood chemistries.

Surgery was uneventful. Five-vessel CABG was carried out where patient remained on cardiopulmonary bypass for 1 hour and 45 minutes and was weaned off without inotropic support, with good hemodynamic profiles.

Post-operatively, he remained hemodynamically stable for 1 hour in intensive care unit, after which he was noted to have excessive bleeding from his tube which was draining the mediastinum. Considering platelet dysfunction, as he was on aspirin previously, six units of random donor platelets were transfused. Within an hour of transfusion, he developed hypotension. His oxygen saturation dropped from 98% to 90%, with PaO_2_/FiO_2 _of 180 and pulmonary artery pressure of 64/39 mmHg. He developed small amounts of frothy, proteinaceous appearing pulmonary exudates from the endotracheal tube that required repeated suctioning. He became increasingly hypotensive and had to be resuscitated per Advanced Cardiac Life Support protocols, but he continued to remain hypotensive. He was rushed to the operating room for exploration of any correctable cause. Due to his hemodynamic instability, he was placed on the heart-lung machine. Doppler ultrasound revealed all five grafts to be functional. Intra aortic balloon pump was inserted and trans-esophageal echocardiography was done to assess left ventricular function. A left atrial line was also placed. Further, six units of packed red blood cells were transfused to combat hemodilution resulting from prolonged second pump run. Despite of all measures, the patient developed frank right heart failure.

Attempts to wean off from machine were unsuccessful and therefore a right ventricular assist device was placed which allowed him to come off heart-lung machine with massive inotropic support. He was shifted to intensive care unit once again but this time in a critical condition with open chest where he was mechanically ventilated. His vital signs continued to demonstrate marginal hemodynamics: blood pressure, 40/10 mm Hg; oxygen saturation, 88% and pulmonary capillary wedge pressure 13–14 mm Hg. There was ongoing loss of clear proteinaceous-appearing fluid from the lungs requiring elevated ventilator parameters to maintain oxygen saturation greater than 90%. The patient continued to desaturate, became increasingly hypotensive with bradycardia. His cardiac output continued to drop as central venous pressure mounted and he could not be resuscitated.

## Discussion

TRALI can be caused by transfusion of any blood product but whole blood-derived platelet concentrates are the most frequently implicated[2]. Our patient also developed respiratory distress soon after platelet transfusion. The risk of TRALI increases if the donor is a multiparous female[3]. Retrospective inquiry of the donors revealed that the donor of the first unit of platelets was a multiparous female.

Patients at increased risk of developing TRALI include those who have hematologic malignancies or underlying cardiac condition[4]. Our patient had undergone cardiac surgery, which predisposed him to develop TRALI, but the exact mechanism underlying this predisposition needs to be explored. Is it hypoxia at the microscopic level that initiates the cascade during cardioplegia?

The pathogenesis of TRALI is complex and there is lack of agreement on any one hypothesis. It can result from both immune mechanisms such as donor derived anti HLA and granulocyte antibodies against recipient's leukocytes or may be non-immune resulting from neutrophil priming agents such as lysophosphotadylcholines and proteins like CD40 ligand which are abundant in stored cellular blood components[5]. The lungs are the main organs affected by the disorder. The reason is its unique microvasculature which allows neutrophils to be sequestered even under physiological conditions. It has been hypothesized that two distinct events are necessary for TRALI: first is recent surgery, severe infection or trauma that not only prime neutrophils but also activate pulmonary endothelial cells resulting in plugging of pulmonary microvasculature by more rigid and less pliable neutrophils; Second is the transfusion of biologically active mediators (anti-HLA or anti-granulocyte antibodies), which activate already primed adherent neutrophils with consequent endothelial damage and capillary leakage[2]. The age of the transfused platelets, plasma and red cells was three, seven and eight days respectively, in our case. These features favor the possibility of both immune and non immune mechanisms to have occurred in our patient. Various laboratory tests are available for detection of antibodies in both patient's and donor sera but the sophisticated techniques coupled with their low sensitivity and specificity have failed to popularize them for diagnostic purpose and their lack of availability in our setting refrained us from doing such work up.

Detection of TRALI in a patient is a diagnostic dilemma and medical personnel should apprehend new onset Acute Lung Injury (ALI) developing during or within 6 hours of blood transfusion[6]. Clinical signs include rapid onset of tachypnea, cyanosis, dyspnea, fever and hypotension. Besides these, certain laboratory criteria need to be fulfilled for establishing diagnosis of TRALI: PaO_2_/FiO_2 _< 300 or pO_2 _<90% at room air in the absence of impaired left ventricular function[1]. Our patient had clinical evidence of hypoxemia as lung auscultation revealed diffuse crackles and decreased breath sounds, while his chest radiographs were characteristic of pulmonary edema. TRALI also needs to be distinguished from anaphylactic transfusion reactions, protamine reactions and transfusion related circulatory overload. Thus, the diagnosis of TRALI is much the matter of skilled clinical judgment, expertise and awareness of clinicians. The significance of the correct diagnosis lies in the fact that most of the patients recover within 24–48 hours without permanent sequel. However, a variable mortality of 11% to 45% has been reported[7]. There are already various concerns regarding the poor short and long term outcomes associated with transfusion of blood products and TRALI associated morbidity and mortality adds to these concerns. Being an under diagnosed condition, these numbers might represent only the tip of an iceberg.

Treatment of TRALI is generally supportive and similar to that for ALI[2]. Aggressive respiratory support, including supplemental oxygen and mechanical ventilation can be life saving as was done in our case also, but unfortunately TRALI proved fatal for him. Other therapeutic measures include extra corporeal membrane oxygenation and additional blood component therapy if clearly indicated. The role of steroids and diuretics that had been previously used has been questioned. We think that the effects of low-dose epinephrine or methoxamine on the pulmonary vasculature and control of blood pressure during and before the occurrence of lung injury needs to be explored as this might revolutionize the management of TRALI.

Since mortality resulting from TRALI can be significant, prevention of the disorder is crucial with the responsibility resting on blood bank services. Haemovigilance data from Scandinavia and the UK demonstrates that utilizing solvent detergent-treated fresh frozen plasma can greatly minimize TRALI probably by dilution and neutralization of leukocyte antibodies and removal of cells and cell fragments in pooled plasma[8]. Similarly, eliminating multiparous females from donating might ensure TRALI prophylaxis by decreasing HLA and granulocyte antibodies in blood products. relevant information not included in the case presentation, and put the case in contextor that explainspecific treatment decisions.

## Abbreviations

TRALI: Transfusion Related Acute Lung Injury; ALI: Acute Lung Injury; CAD: Coronary artery Disease; CABG: Coronary Artery By-pass Surgery; CD: Cluster of Differentiation; HLA: Human Leukocyte Antigen.

## Consent

Written informed consent was obtained from the patient's daughter for publication of this case report.

A copy of the written consent is available for review by the Editor-in-Chief of this journal.

## Competing interests

The authors declare that they have no competing interests.

## Authors' contributions

FAB and HS both analyzed and interpreted the patient data regarding the hematological event and wrote the manuscript. HS was the surgeon of the patient reported. Both authors read and approved the final manuscript.
